# Education and Training: Key Factors in Global Occupational and Environmental Health

**DOI:** 10.29024/aogh.2328

**Published:** 2018-08-31

**Authors:** Roberto G. Lucchini, Melissa McDiarmid, Gert van der Laan, Mitchel Rosen, Donatella Placidi, Katja Radon, Mathuros Ruchirawat, Lena Kurtz, Philip Landrigan

**Affiliations:** 1 Icahn School of Medicine at Mount Sinai, New York, US; 2 Occupational Medicine, University of Brescia, IT; 3 Division of Occupational and Environmental Medicine, University of Maryland School of Medicine, Baltimore, US; 4 Learning and Development of Occupational Health Foundation, NL; 5 Office of Public Health Practice, Rutgers School of Public Health, Piscataway, New Jersey, US; 6 Center for International Health at Institute for Occupational, Social and Environmental Medicine, University Hospital Munich (LMU), Munich, DE; 7 Chulabhorn Research Institute, Bangkok, TH

## Abstract

**Introduction:**

Education and training in Occupational and Environmental Health (OEH) play an important role in building global capacity and contribute to safer working conditions. The shortage of occupational health professionals, the lack of knowledge and a high number of occupational accidents and diseases stress the demand for providing further education and training in OEH. This need is especially urgent in low and middle-income countries. Three international courses on OEH provide valuable insights on how to develop successful trainings in the field and how to contribute to the creation of healthy and safe workplaces.

**Methodology and Results:**

The courses “Global Occupational and Environmental Determinants of Diseases: a multidisciplinary and multicultural approach for prevention” (University of Brescia, Italy), “Advanced International Training Course in OEH” (Chulabhorn Research Institute, Bangkok, Thailand) and “Teaching interventions crossing borders” (Ludwig-Maximillians-Universität, Munich, Germany) offer a unique networking opportunity for health professionals from low-, middle- and high-income countries. Three main characteristics of learning were identified as successful for adult learning in OEH: Interdisciplinary learning allows exchanging skills and knowledge and therefore closes gaps between the several disciplines within OEH. Experiential learning enables participants to reflect on their personal experiences, e.g. through workplace visits. Finally, active learning empowers the learner to take the leading role in the learning process using e.g. problem- or project-based learning approaches.

**Conclusion:**

Training and education in OEH should become a higher priority by including it in the standard public health and medical curricula. For this, train the trainer approaches are essential in many countries. Any OEH training should follow the interdisciplinary, experiential and interactive learning approach and should be accessible to participants from all regions.

## New global courses on Occupational and Environmental Health

Education and training of health workers and clinician partners play an enormous role in the success of any prevention practice effort in the public health arena. Younger generations of professionals are necessary to sustain the gains current practitioners have achieved and this need is acutely felt in the fields of occupational and environmental health particularly in its global perspective.

Even as the World Health Organization estimates that 24% of the global burden of disease and death is attributable to occupational and environmental exposures [1], a severe global shortage exists in the number of health professionals trained in environmental and occupational health (OEH). This shortage is especially acute for occupational physicians [2] and is likely to worsen in future years with a globally growing working population, steady increases in chemical production, and increasing global demand for safer workplaces and healthier environments [3].

Education and training are critically important for building global capacity in OEH. Training is needed to enhance the knowledge, skills, and practice of the current workforces in these fields and to build the future generation of workers. Working professionals, both those trained in OEH and those serving in roles without formal specialty instruction, need training to improve safety and health practices, design safer workplaces and provide cleaner environments.

This Special Issue of the Annals of Global Health is the second volume of a series of three international, multi-disciplinary recurring courses on Occupational and Environmental Health:

1. “Global Occupational and Environmental Determinants of Diseases: a Multidisciplinary and Multicultural Approach for Prevention,” University of Brescia, Italy [4].
2. “Advanced International Training Course in Occupational and Environmental Health,” Chulabhorn Research Institute, Bangkok, Thailand [5].
3. “Teaching Interventions Crossing Borders,” at the Ludwig Maximillian University of Munich, Germany [6,7].

In addition to high-quality, hands-on training in occupational and environmental health, these courses provide unique opportunities for networking among students and professionals from both public health and medical specialties, from high-income and low and middle-income countries, and for developing multi-national approaches to continuing education that extend beyond the courses. Students and faculty attending the courses are encouraged to provide information about occupational and environmental health in their countries, including topics such as Occupational Safety and Health (OSH) services coverage and organization; workers’ benefits and compensation; ratification and implementation of policies from the International Labour Office (ILO) and the World Health Organization (WHO); preventive interventions in environmental health; and remediation of hazardous waste sites. The Munich course is additionally focused on another important task of the OSH experts: how to train workers, managers, and the community, as well as healthcare and safety professionals and political stakeholders. Course participants learn how to set up a competency-based training program for adult learners, according to a ‘train the trainer’ approach.

Active learning methods are offered by all three courses and thus provide opportunities for networking and bi-directional exchange of ideas and experiences between faculty and participants. This was mainly achieved by the structure of the courses allowing ample time for groupwork, discussion, and presentation by participants. Education and training programs provide highly valuable outputs when participants are actively involved, are encouraged and have opportunities to illustrate data and critical policy aspects from their personal experience or work on a concrete problem (problem-based-learning). This is especially relevant for occupational and environmental health, given the extreme lack of information and research data from the global perspectives, the need for education [8] and capacity building [9].

The LDOH Foundation (Learning and Development of Occupational Health) has the mission to support professionals through developing and promoting quality education and information on Occupational Safety and Health. Influenced by our three courses, LDOH started supporting health professionals through developing and promoting good quality education and information on Occupational Safety and Health. A library of online and blended lessons, modules and courses from all over the world is available at the LDOH website (https://ldoh.net/). LDOH is involved in two EU-funded projects focusing on capacity building for Occupational and Environmental Health. The first takes place in Turkey, where the Ministry of Health has implemented the project Scientific Performance of Public Health Institution of Turkey (ESPrIT – http://esprit-ohs.eu/en/), funded by the EU Horizon2020 program. ESPrIT includes a one-week training module in occupational health surveillance to illustrate the principles of adult learning: goal oriented, practical and problem centred. Step by step information (20-minute presentations) is provided on the different phases of a surveillance project and small interdisciplinary groups work on a mutually chosen subject. The other is an Erasmus+ programme coordinated by the University of Milan, Italy, jointly with ten universities in Central Asia and India in which educational materials for a blended learning Master Program in Occupational and Environmental Health are developed.

This Special Issue is an example of what participants can produce to fill the knowledge gap about the global context, by providing data on specific situations in the many parts of the world from which they come. This data is absolutely necessary to provide more accurate estimates on the global burden of occupational and environmental diseases and to optimize preventive strategies. Data available today is almost completely related to Higher Income Countries [10–11] and do not reflect accurately the current trends in most countries of the world.

According to a recent survey by the International Occupational Medicine Society Collaborative on Global Trends in Occupational Medicine [2], the number of occupational physicians is not sufficient to meet demand. This trend is likely to worsen with increased demand and fewer trainees [3]. In addition, legislative and government support for occupational medicine, while present in some countries, is not universal, due to a lack of understanding by employers and government officials of the benefits of an occupational medicine service. These two elements (shortage of professionals and lack of knowledge) clearly indicate the severe need for education and training in Global Occupational and Environmental Health. This discipline can also attract and offer potential for career development for students from Higher Income Countries, who are showing increasing interest in Global Health and may be attracted to the Occupational and Environmental field.

## Scope of the Discipline and Statement of Need

Occupational safety and health is a broad field that addresses critical issues to help ensure workers and work places remain safe. No matter the profession, specific job or workplace, there are hazards present that need to be controlled. In the US, there were 4,836 fatal work injuries [12] and approximately 2.9 million nonfatal workplace injuries and illnesses in 2015 [13]. Globally, it is estimated that 2.3 million workers are killed each year (including 352,769 fatal work injuries and almost 2 million from occupational diseases) and there are over 313 million non-fatal occupational accidents [14]. The burden is particularly heavy in low and middle-income countries, where manufacturing is mostly concentrated and health and safety law and its application is often not implemented properly [15].

In the United States, the Occupational Safety and Health Act was enacted to “assure safe and healthful working conditions for working men and women [16].” The European Union Strategic Framework on Health and Safety at Work 2014–2020 lists the goal of “ensuring a safe and healthy work environment for over 217 million workers in the EU [17].” Other countries have enacted safety and health legislation to enhance safety and protect workers, with much of the legislation included in country profiles on the ILO website [18].

There is a critical need for education and training in occupational safety and health to address basic and more advanced threats to worker health. Training is needed for the current and future OSH workforce. Working professionals, trained in OSH, together with those professionals working in roles potentially related to OSH, may gain capacity through short-courses and collaborate with OSH trained professionals to improve safety and health practices and design safer workplaces. Training the future occupational health workforce will enable the practices and actions taken to date to be sustained and to be built upon for the benefit of future generations.

## Interdisciplinary, Experiential and Active Education in Occupational Safety and Health

Educators need to look at the methods and practices used to provide occupational safety and health training. OSHA standards provide minimal guidance for training content and delivery methods [19]. Developing and implementing training programs is difficult, as OSH is a broad field and encompasses many disciplines including, among others, occupational medicine and nursing, industrial/occupational hygiene, occupational safety, ergonomics, and occupational psychology. The focus of OSH practice is the worker or workplace, and each of these disciplines focus on a portion of the overall field of OSH. Industrial/occupational hygiene looks at worker exposures, occupational safety identifies workplace injury hazards, and occupational medicine examines health threats to workers and clinical care when they are injured or ill. Training must include how each of these distinct disciplines overlap and intersect.

### Interdisciplinary Learning

OSH training should be interdisciplinary and include experiential learning as the basis for the development and implementation of training programs. This interdisciplinary approach will bridge the gap between the OSH disciplines, and refine the focus of OSH practice on improving the health and safety of workers and reducing hazards in the work place. Interdisciplinary training programs also provide opportunities for trainees to present their professional and country-based experience of a topic, thus improving their skills and knowledge base across disciplines. Erickson [20] writes about “interdisciplinarity” as a means to increase safety performance. Interdisciplinarity “integrates knowledge from different disciplines. It blends the assumptions and practices of each into an integrative relationship to accomplish a larger purpose such as improving safety performance.” Recognizing the importance of this approach, the National Institute for Occupational Safety and Health (NIOSH) provides extramural funding to support Education and Research Centers (ERCs) to provide high-quality, interdisciplinary graduate training, research training, continuing education, and outreach in the core occupational safety and health disciplines [21]. The ERCs are model training programs that are based on the understanding and awareness of the interdisciplinary nature of OSH professional practice.

### Experiential Learning

Incorporating experiential learning into training programs provides valuable opportunities for trainees to visualize occupational hazards. The experiential learning theory (ELT) defines learning as “the process whereby knowledge is created through the transformation of experience. Knowledge results from the combination of grasping and transforming experience [22].” Experiential learning is a cycle that includes experiencing, reflecting, thinking, and acting. Training developed with the ELT identifies why what students are learning is important, it allows participants to practically navigate through content, and it uses real life examples and scenarios to anchor instruction. Experiential learning connects prior knowledge with new knowledge [23], allowing trainees to reflect on their personal experiences to transform the way they understand and act on what they learned.

The New York and New Jersey ERC (NYNJ ERC) developed their Historical Perspectives on Occupational Safety and Health course in 2006 to provide trainees with interdisciplinary, experiential learning opportunities. In the Historical Perspectives course, trainees visit workplaces to experience how workers work and experience the occupational hazards and controls at work sites. These work place visits are a highlight of the academic training provided by the NYNJ ERC. Trainees’ comments on the course exemplify the value of interdisciplinary and experiential learning. Several direct quotes from the evaluation from the Historical Perspectives course include:

- “How crucial inter-professional education is to become a good, competent occupational medicine physician. Seeing workers at their own sites, helps you learn and appreciate their work conditions, their challenges and hazards on a whole new level that can never be learned as well from a classroom or clinic.”
- “This experience opens my eyes to question conditions of fellow workers, to ask more informed questions about what is being done to prevent not only injury in the work place but what can we do to systematically improve and prevent injury and promote well-being in the first place.”
- “Workers may not feel empowered to advocate for themselves if an employer asks them to complete tasks that are not compliant with the restrictions. This is something that I will keep in mind. With the knowledge that I am gaining through experiences like this tour I will be able to advocate for workers.”
- “The more powerful aspect of learning comes from experiencing things first hand, and from relevant ‘people,’ not just teachers. That kind of learning goes deeper and lasts longer.”

### Active Learning

Based on the constructivism learning theory, when developing (adult) learning programs in OSH, as in any other discipline, learning is considered as an active process controlled by the learner and based on his/her previous experiences [24]. Such experiences always differ from one person to another but are even more pronounced among participants from high, middle and low-income countries like those attending these courses. Therefore, in the setting of the described summer courses it was essential to take this previous experience into account. It also has to be acknowledged that rather than just listening to the lecturer, learners remember more when they interact with each other and work on real and relevant topics [25,26]. Furthermore, thanks to current technology, every learner has all information right at hand. Therefore, the most important task of the teacher is to act as a facilitator and accompany the learner to find, understand and apply current evidence [25,27].

Based on theoretical considerations, the Munich summer course uses a very interactive problem and employed a project-based learning approach during which participant develop a teaching intervention for their workers, managers, and community, targeting a concrete OSH problem in their home country. After successfully formulating “smart” (Specific, Measurable, Attainable, Relevant, Timely) learning objectives for the training, participants approach a teaching scheme structured in five phases of learning: 1) Adjusting and initial setting of the ‘tone’ and best mood for learning; 2) Reactivation of learners’ previous knowledge; 3) Information about the new knowledge; 4) Processing of the new information; 5) Evaluation, according to the ‘ARIPE’ (Adjust, Reactivate, Inform, Process, Evaluate) model [28]. The ARIPE steps follow and support the learning process [28]. For each ARIPE-step, participants become familiar with several interactive methods that can be applied within the different social forms of learning and that ensure an active role of the target group. Participants in the Munich course directly apply the ARIPE structure with the interactive methods to the teaching intervention they develop. In order to encourage exchange, participants work in small groups of up to four students, supported by tutors. Back home, participants apply their teaching intervention to their target groups and evaluate the outcomes.

### Models for Education and Training

Based on the models of interdisciplinary, experiential, and active learning, all three training courses incorporate site visits, including visits to a marble quarry and milling operations, a steel plant and an automobile parts manufacturing facility in Italy, to the Ramathibodi Hospital, the largest health care center in Bangkok, and to a large car manufacturing facility in Germany. The addition of these workplace visits enable the participants to understand the complexities of work, experience how work impacts worker safety, and identify ways to control workplace hazards. Based on trainee feedback, the inclusion of these work place visits is a valuable learning experience. One trainee commented that he “gained practical workplace insight and experience” from the visits to the workplaces. Another commented, “The interdisciplinary approach helps us to resolve various issues and come up with common solutions that will benefit the workforce.”

The model used by the NYNJ ERC and the Summer Programs is not new. Over 300 years ago Bernardino Ramazzini visited workplaces to observe how various types of work were performed and to discuss illnesses with workers [29,30]. In 1910 Alice Hamilton investigated the “dangerous trades” in Illinois, visiting workplaces to see the conditions faced by workers and to understand the hazards present that were making workers ill [31]. The concept of experiential learning in OSH, that you need to see the hazards and experience what workers are facing, is the core concept that is incorporated in the design of the programs described.

Utilizing interdisciplinary and experiential learning is a powerful way to provide information, because the learners experience the conditions faced by workers. The programs offered by the NYNJ ERC, the Brescia Summer Program, the Bangkok International training course and the Munich Summer School are examples and models that can be utilized by others across the globe. By experiencing the work places, trainees are able to understand the value of interdisciplinary OSH teams, and working collaboratively ultimately improve health and safety conditions for workers and work places.

## Recommendations

- Given the deficit of specialist expertise in the field globally, education and training in Occupational and Environmental health must be given a greater priority at the national, regional and global level.
- Capacity building as a priority may be achieved through a ‘Train the trainer’ approach, where the target groups plays an active role.
- Courses should be intensified and offered more extensively also to generalists in clinical medicine and public health, covering all geographical areas and with special focus on sectors with high occupational and environmental hazards.
- Training should be fully interdisciplinary using an active, experiential learning approach.
- Aspects of Occupational and Environmental health should be integrated into standard public health and medical curricula when possible, to optimize human resources and capacity building.

This Special Issue of the Annals of Global Health will show-case the professional efforts of the participants of the three international courses described above. While some participants had occupational health training and are authors here, the majority were generalists in clinical medicine or public health, who were engaged in occupational/environmental health activity in their home countries, as a ‘collateral’ assignment. Their documentation of local problems and solutions from their countries, described here, are first-hand “reports from the field” and help to close the existing knowledge gap between countries, regarding the occupational health agenda globally.
